# Day 4 Versus Day 5 Fresh Embryo Transfer in In Vitro Fertilization: Is It All About Timing?

**DOI:** 10.3390/jcm14134596

**Published:** 2025-06-28

**Authors:** Alper Şişmanoğlu, Süleyman Cemil Oğlak, Cenk Özcan, Ulun Uluğ

**Affiliations:** 1 Department of Obstetrics and Gynecology, Altınbaş University Faculty of Medicine, 34147 Istanbul, Türkiye; alper.sismanoglu@altinbas.edu.tr; 2 Department of Obstetrics and Gynecology, Health Sciences University, Gazi Yaşargil Training and Research Hospital, 21500 Diyarbakır, Türkiye; 3 Department of Embryology, Ota & Jinemed Hospital IVF Center, 34357 Istanbul, Türkiye; cenko@otajinemedhastanesi.com; 4 Department of Obstetrics and Gynecology, Haliç University Faculty of Medicine, 34060 Istanbul, Türkiye; uulug@hotmail.com

**Keywords:** blastocyst stage embryo transfer, embryo quality, fresh embryo transfer, in vitro fertilization

## Abstract

**Objective:** Most studies concentrate on comparisons between the cleavage stage and blastocyst stage of embryos during in vitro stimulation treatment. We aimed, in this study, to compare the pregnancy rates of day 4 or day 5 blastocyst transfers, all derived from fresh, antagonist-regulated in vitro fertilization (IVF) cycles, and to evaluate the factors affecting pregnancy success. **Methods:** This retrospective cohort study evaluated 3681 fresh embryo transfer cycles conducted at a private IVF center between 2019 and 2021. Patients were divided into two groups based on the day of embryo transfer: day 4 (Group 1) and day 5 (Group 2). Subgroup analyses were performed according to age (≤40 vs. >40 years) and the number of oocytes retrieved (≤4 vs. >4). All patients underwent ovarian stimulation with FSH alone or in combination with hMG, and GnRH antagonist protocols were used for pituitary suppression. Final oocyte maturation was triggered with recombinant hCG, and fertilization was achieved via intracytoplasmic sperm injection (ICSI) for all cases. Embryos were cultured in sequential media and assessed daily until transfer on day 4 or day 5, based on embryo morphology and clinic logistics. **Results:** Pregnancy was more likely among women under 40 than among women over 40. There were a total of 1217 women who underwent day 4 transfer and 2464 women who underwent day 5 transfer. A total of 660 (54.2%) of the women transferred on day 4 developed pregnancy. Among those transferred on day 5, 1610 (65.3%) developed pregnancy. When compared to the 4th day, a single embryo transfer on the 5th day enhances pregnancy success by 1.8 times, while two embryo transfers raise it by 1.6 times. Furthermore, when the number of oocytes is greater than four and the number of embryos transferred is two, the pregnancy success rate is 2.5 times higher when embryo transfer is performed on the fifth day versus the fourth day. Regardless of age, oocyte count, or number of embryos transferred, 5th-day fresh embryo transfers enhanced pregnancy success by 1.9 times compared to 4th-day transfer. **Conclusions:** Transfers of fresh embryos on day 5 are superior to those on day 4 and should be favored, especially for people over the age of 40, regardless of the number of embryos transferred, even if that individual has fewer than four oocytes.

## 1. Introduction

Infertility, which is defined as the inability to conceive after at least one year of unprotected sexual activity, affects approximately 20% of couples worldwide. More than 2.5 million treatment cycles are carried out on people every single year throughout the whole world, and more than 8 million children have been born as a result of IVF [1]. The effectiveness of IVF depends on the overall success rate of the treating clinic and the characteristics of the infertile couple. Accordingly, the factors that are thought to affect the success of IVF include the physical environment, genetics, psychological factors, serum levels of hormones (Anti mullerian hormone, follicle stimulating hormon, luteinizing hormone, estradiol, progesterone), sperm and egg characteristics, the couples’ age and body mass index [2]. It has been shown that using ovarian stimulation (OS) to promote the formation of multiple follicles is the most effective method for improving the success rate of IVF [3].

Sustaining embryo–endometrial synchronization is crucial for optimizing clinical outcomes subsequent to embryo transfer. The primary explanation for this is the occurrence of a clinical “window of implantation” (WOI) of the endometrium, which is thought to be optimal for embryo implantation [4]. The rate of embryo implantation is dramatically lowered when the embryo and endometrium are asynchronous, according to a recent study comprising hundreds of transfers in Europe and the United States [5]. It has been years since it was accepted that blastocyst transfers are superior to transfers at the cleavage stage and promise better pregnancy rates, and now, 5th-day transfers are always included in the IVF routine in most IVF centers. This was also the truth for single-embryo transfers aiming to reduce multiple pregnancies, and Papanikolaou et al.’s study on patients younger than 36 years of age in 2006 also documented greater success in 5th-day transfers [6,7]. Meta-analysis stating that the best option for optimal pregnancy rates in those patients with the same number of transferred embryos was to transfer them at the blastocyst stage rather than the cleavage stage [8]. The available literature lacks adequate data on the transfer of blastocysts on the fifth day. In addition, as stated in the introduction, several in vitro fertilization centers deliberately or inevitably conduct certain transfers on day 4 instead of day 5 due to the coinciding of the blastocyst stage with weekends or holidays [9]. As far as we know, there has been no study conducted on a significant scale that compares these two stages of embryo development in relation to the outcomes of pregnancy. Therefore, we think that our study is important because of its high volume and presenting data on an unclear subject.

The aim of this study was to compare the clinical pregnancy rates of day 4 or 5 blastocyst transfers, all derived from fresh antagonist-regulated IVF cycles, and to evaluate the factors affecting pregnancy success.

## 2. Material and Methods

In this retrospective cohort study, we took into account 3681 antagonist fresh embryo transfer cycles performed at a private IVF center from 2019 to 2021 year. We organized the patients in to two groups: Group one: day 4 transfers; and Group 2: day 5 transfers. Groups were investigated according to embryo transfer day, number of oocytes collected, number of fertilized eggs and number of embryos transferred. In addition, subgroups were formed based on age: 40 years of age and younger, over 40 years of age, and those for whom the number of oocytes collected was ≤4 or >4.

Individuals experienced ovarian stimulation with FSH alone or in conjunction with hMG. For pituitary suppression, GnRH antagonists, either ganirelix acetate (Orgalutran 0.25 mg/0.5 mL, MSD, Ravensburg, Germany) or cetrorelix (Cetrotide 0.25 mg, Merk Serono, Idron, France), were used in all cycles included in this study. Final oocyte fertilization was accomplished with recombinant human chorionic gonadotropin (Ovitrelle 250 micrograms, Merk Serono, Modugno (BA)/Italy) 35–36 h before the ovum pick-up procedure. The embryos were fertilized via intracytoplasmic sperm injection (ICSI). ICSI is a routine fertilization option in our laboratory regardless of the reason for infertility and we perform it on all patients. ICSI was done 2–4 h after oocyte retrieval. The embryos were cultured in sequential media. Injected oocytes were incubated in groups (3 or 4 oocytes in one drop) in 40 μL culture medium droplets (Sydney IVF cleavage medium, Queensland, Australia) under mineral oil (Sage; oil for tissue culture, Trumbull, CT, USA). On day 1, 16–20 h after injection, oocytes were examined for the presence of two pronuclei. At day 2 (41–44 h after injection) embryos were scored for the number and the size of blastomeres (equal or unequal (>25% difference in size)), the degree of fragmentation. Embryos were then rinsed and transferred to individual 40 μL droplets of G-TL medium (Vitrolife. Gothenburg, Sweden) under mineral oil and further individual development was recorded daily until day 4 or day 5. After selection of the best embryo/embryos based on morphological characteristics, embryo transfers were performed on day 4 or day 5. We assessed the embryo qualities and choose the best embryo/s according to the 2011 ESHRE Istanbul Consensus for D4 embryos [10].

The transfer of selected blastocysts occurred on the fourth or fifth day of fresh cycles. Blastocysts were examined in a number of different ways. In fresh blastocysts, the developmental stage, blastocyst diameter, ICM dimensions, and trophectoderm cell count were recorded. In addition, the presence or absence of necrotic foci in the ICM, the degree of ICM compaction, and the presence of sparse areas in the trophectoderm were recorded. The selection of blastocysts for transfer was based on a subjective combination of these observations. There was some preference for transferring on day 4, if healthy blastocysts were present. This preference coincided with the weekend, official or religious holidays, or the day of departure for patients, who were traveling from another country. Despite everything, there is no clear evidence to choose the 4th or 5th day after the ICSI. There were some doctors who preferred a transfer on the 4th day because they were afraid that they would be there on 5th day and wanted to complete the transfer phase on the 4th day of embryo development.

Patients were administered 100 mg of IM Progesteron in oil daily or Micronized Progesterone suppositories (600–800 mg/day starting from the oocyte pick up day untill the end of 10th week of pregnancy. It is important to know that synchronized development of healthy embryo and a receptive endometrium is critical for successful implantation to take place. Progesteron supplementation either by intramuscular or vaginal route is a mandatory in all cycles especially in fresh cycles to obtain the synchrony between the endometrium and the embryo. All transfers were with embryos from fresh autologous cycles and performed under the guidance of transabdominal ultrasound. Pregnancy was defined as positive if blood hCG levels exceeded 5 mIU/mL 11–12 days after the embryo transfer. Clinical pregnancy was explained by the detection of one or more gestational sacs during a subsequent transvaginal ultrasound examination at approximately 5–6 weeks of gestation, following the detection of increasing hCG titers. In this investigation, clinical pregnancy rates (PR) were reported per transfer. Informed consent was obtained from all individuals. Ethics committee approval was obtained from the Altinbas University Ethics Committee (04.09.2023-56441).

## 3. Statistical Analysis

The SPSS 25.0 (IBM Corporation, Armonk, New York, NY, USA) program was used in the analysis of the variables. The conformity of the data to the normal distribution was evaluated with the Shapiro–Wilk francia test. The Mann–Whitney U test was used with Monte Carlo results to compare the variables of age (female), age of spouse (male), number of oocytes, number of M2 oocytes, mature egg ratio, fertilization rate and number of embryos transferred according to pregnancy, which were not normally distributed. The Pearson Chi-Square test was used to compare the categorical variables by pregnancy, while the Fisher Exact test was used with Monte Carlo simulation results when the expected value was less than 5. Column ratios were compared and expressed as the Benjamini–Hochberg corrected *p* value results. According to the results of this analysis, the odds ratio (OR) 95% confidence interval was used to show how many times the transfer day was higher than the pregnancy rate. According to the pregnancy dependent variable, age was adjusted according to the oocyte count and transferred embryo count variables, and a logistic regression test was used to determine the cause-effect relationship according to the explanatory variable of the transfer day. While quantitative variables were expressed as mean (standard deviation) and median (1st Quartile/3rd Quartile) in the tables, categorical variables were shown as n (%). The variables were analyzed at a 95% confidence level, and a *p*-value of less than 0.05 was considered significant.

## 4. Results

According to pregnancy status, Table 1 compares age (female), spousal age (male), oocyte count, M2 oocytes, mature egg ratio, fertilization number, and transferred embryo number. Pregnancy was more prevalent among women under 40 than among women over 40. (*p* < 0.001) The number of pregnancies among patients who underwent embryo transfer on the fifth day was statistically substantially higher than among those, who underwent embryo transfer on the fourth day. (*p* < 0.001) The reported pregnancy rates may appear unusually high. This is partly due to the nature of the patient population at our clinic. Patients included in this study were selected based on good ovarian response, absence of uterine pathology, and no severe male factor infertility. Thus, our cohort represents a relatively favorable subgroup in IVF practice. In terms of pregnancy success, female age, male partner age, oocyte count, M2 oocyte count, and the number of transferred embryos were significant, but mature oocyte count and fertilization rate were not.

The results of comparing pregnancy status based on age, number of oocytes, and number of transferred embryos on the day of embryo transfer are presented in Table 2. Day 5 embryo transfer had higher pregnancy success rates than day 4 embryo transfer for women under 40 with more than 4 oocytes and 1 or 2 embryos transferred (*p* < 0.001). When the number of oocytes is greater than four and the number of embryos transferred is one or two, embryo transfer on the fifth day increases the likelihood of a successful pregnancy by 1.6 times more than embryo transfer on the fourth day (*p* < 0.001). One embryo transfer on the 5th day increases pregnancy success by 1.8 times compared to the 4th day, whereas two embryo transfers increase pregnancy success 1.6 times. (*p* < 0.001) There was no difference in pregnancy success when the total number of oocytes was less than four in women over the age of 40, regardless of the number of embryos transferred. (*p* = 0.999 and *p* = 0.090). However, when the number of oocytes was greater than four and the number of embryos transferred was two, pregnancy success was substantially greater when the embryo transfer was performed on the fifth day as opposed to the fourth (*p* < 0.001). Moreover, when the number of oocytes is greater than four and the number of embryos transferred is two, the pregnancy success rate is 2.5 times higher when embryo transfer is undertaken on the fifth day compared to the fourth day (*p* < 0.001).

A total of 339 patients with 4 or fewer oocytes were transferred on the 4th day, and pregnancy developed in 36 (29%) women with 1 embryo transfer, while pregnancy did not occur in 88 (71%) women. In those with 2 embryo transfers, 99 (46%) developed pregnancy and 116 (54%) did not. In the group with more than 4 oocytes, pregnancy occurred in 204 (60.2%) women with 1 embryo transfer, but not in 151 (39.8%) women. In the group with 2 embryo transfers, pregnancy occurred in 261 (49.9%) and did not occur in 262 (50.1%).

**Figure 1:**
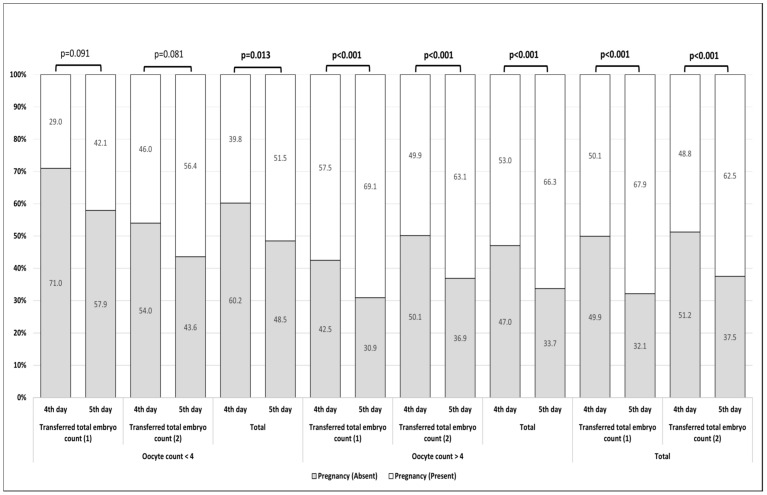
Comparison of pregnancy success rates.

A total of 167 people with more than 4 oocytes were transferred on the 5th day and pregnancy developed in 24 (42.1%) women with 1 embryo transfer, while pregnancy did not occur in 33 (57.9%) women. In those with 2 embryo transfer, 62 (56.4%) developed pregnancy and 48 (43.6%) did not. In the group with more than 4 oocytes, pregnancy occurred in 856 (69.1%) people who had 1 embryo transfer and did not occur in 383 (30.9%) people. In the group with 2 embryo transfers, pregnancy occurred in 1524 (66.3%) but not in 773 (33.7%). In the group with 1 embryo transfer on day 5, pregnancy occurred in 880 (67.9%) but not in 416 (32.1%). (Table 3) In the group with 2nd embryo transfer, 730 (62.5%) developed pregnancy while 438 (37.5%) did not. In Table 3, regardless of age, the effects of oocyte count and transferred embryo count on pregnancy success are compared. When the number of oocytes was less than four, there was no difference in pregnancy success between transferring one or two embryos (*p* = 0.091, *p* = 0.080) (Figure 1).

If the number of transferred embryos is one or two and the number of oocytes is greater than four, pregnancy success is substantially higher on day 5 compared to day 4. (*p* < 0.001)

The findings of the test that determined the cause-effect relationship according to the explanatory factors of the day of transfer, age, number of oocytes, and number of embryos transferred are presented in Table 4. The findings of the logistic regression study indicate that embryo transfer on the fifth day results in a 1.9 times greater chance of successfully achieving a pregnancy.

## 5. Discussion

The total number of transferred embryos and the day of embryo transfer according to embryo development have a substantial effect on IVF success and the risk for complications such as multiple pregnancies and ovarian hyperstimulation syndrome. There are contradictory perspectives on this issue in the literature. A study comparing single-embryo transfer and multiple-embryo transfer found that single embryo transfer resulted in a reduced rate of multiple births, but there was no significant difference in the rate of live births [9,11]. In contrast, a meta-analysis revealed that the live birth rates for single-embryo transfer were lower than those for multiple-embryo transfer [12]. In our study, it was statistically demonstrated that where the number of transferred embryos was 1 or more, there was an increase in the pregnancy success rate. While transferring one embryo increased the chance of getting pregnant by 2.1 times, transferring two embryos increased the chance of getting pregnant by 1.8 times in patients with a bad ovarian response—defined as obtaining ≤ 4 oocytes during the oocyte pick up day.

The empirical evidence suggests that the day of embryo transfer in IVF procedures exerts a significant impact on the rates of successful pregnancy outcomes. Our study revealed that even when factors such as weekends, doctors’ holidays, or the patient’s need to depart were taken into consideration when deciding on the transfer day, the optimal day for transfer was found to be the fifth day. There are fewer studies on embryo transfer on day 4, as embryos are typically transferred on day 3 (cleavage stage) or days 5–6 (blastocyst stage). However, the current literature contains a few investigations on day 4 transfers and pregnancy rates: According to studies, day 5–6 transfers result in greater live birth rates than day 2–3 transfers. Blastocyst transfers are regarded as superior due to their superior embryo selection and scheduling, which is more compatible with the endometrium [13]. The majority of these studies do not specifically evaluate embryo transfers on day 4 and pregnancy rates, but they do demonstrate that day 4 embryos can be used successfully in certain situations. This demonstrates the potential of day 4 embryo transfer in terms of embryo development and endometrium compatibility. In our study, it was determined that transferring embryos on the fifth day increased pregnancy success by 1.9 times compared to transferring embryos on the fourth day, regardless of the effects of age, oocyte count, and the number of embryos transferred, all of which influence pregnancy success. The literature demonstrates that day 5 transfers have higher success rates than day 6 transfers, although it is difficult to draw definitive conclusions about day 4 transfers. The success of day 4 embryo transfers will depend on the quality of the embryo, the condition of the endometrium, and the patient’s particular circumstances. It is possible that the influence of oxygen concentration on embryo development and transfer timing contributed to this outcome [14]. Irrespective of the reason for transfer on the 4th day (medical or otherwise), 5th day transfers always enjoyed a higher success rate in our study. Briefly, additional research is required to elucidate the success of embryo transfers on day 4.

According to the literature, day 5 transfers appear to enjoy a greater success rate. This situation can be attributed to the fact that day 5 transfers allow for a prolonged period of observation of embryo development. This allows for the selection and transmission of embryos that are healthier and more developed. In addition, embryos at the blastocyst stage have a greater chance of implantation and a reduced risk of genetic abnormalities. Embryo-endometrial synchronization is critical for implantation success as it is stated in a study by Teh WT et al., 2016 [5]. Transfer on day 5 aligns better with the ‘window of implantation’, when the endometrial receptivity is optimal. Furthermore, day 5 blastocysts typically have undergone embryonic genome activation, increased cell numbers, and superior morphology, all associated with higher implantation potential and lower aneuploidy risk. In a systematic review and meta-analysis, it is stated that day 4 transfers are as successful as day 5 transfers and healthy pregnancy can be obtained in embryos that can reach 4 days. However, in the articles considered in this study, different types of mediums, embryo classifications and numbers of transferred embryos were used, which decrease the value of this meta-analysis [15]. Another study by Holschbach et al., 2017, states that day 4 embryo transfers are equally successful compared to day5 embryo transfers [16]. In a study by Glujovsky D et al., in 2022, it is also stated that they are uncertain that cleavage stage embryo transfers improve the clinical pregnancy rates [17]. Also, the PRECiSE trial by Neuhausser WM et al., 2020 is being conducted to show that blastocyst transfers are not superior to cleavage stage embryo transfer in low ovarian reserve patients [18]. In order to evaluate the efficacy of 4th day transfers, it is crucial to consider additional research and patient-specific factors. Day 4 transfers were not based on clinical indication but often due to logistical constraints such as weekends, public holidays, or scheduling issues. This non-random selection introduces potential bias when comparing outcomes of day 4 vs. day 5 transfers.

The limitation of our study is that we are unable to evaluate and demonstrate additional patient clinical characteristics, such as hormone levels, the presence of comorbid diseases, body mass index, etc. The fact that it was a single-center study was another limitation of our study. Additionally, it is important to recognize that day 4 blastocysts represent a subgroup of embryos that develop faster and are likely of high developmental competence. These embryos may have produced similar or better outcomes if transferred on day 5, thus the clinical interpretation of their lower success needs cautious evaluation.

While our results support day 5 transfer, they must be interpreted within the limitations of a retrospective design. Day 5 transfers may benefit from better embryo selection and timing with endometrial receptivity, but decisions should be individualized based on patient needs and clinical context.

## 6. Conclusions

In conclusion, we may say that in our group of patients, day 5 transfers had higher success rates than day 4 transfers, regardless of the number of embryos transferred, even if that individual was older than 40 and had fewer than four oocytes. However, it is essential to evaluate the situation of each patient to determine the most appropriate treatment options for each patient.

## Figures and Tables

**Table 1:** Basic characteristics of the participants.

	Pregnancy			*p*
	Total	Absent	Present	
	n (%)	n (%)	n (%)	
**Age (female)**				**<0.001 ^c^**
≤40	3188 (86.6)	1155 (78.5)	**2033 (92)**	3.1 (2.6–3.8) ^OR^
>40	493 (13.4)	**316 (21.5)**	177 (8)	
**Transfer day**				**<0.001 ^c^**
4th day	1217 (33.1)	**617 (41.9)**	600 (27.1)	1.9 (1.7–2.2) ^OR^
5th day	2464 (66.9)	854 (58.1)	**1610 (72.9)**	
	**Median (Q1–Q3)**	**Median (Q1–Q3)**	**Median (Q1–Q3)**	
**Age (female)**	34 (29–38)	35 (31–40)	33 (29–36)	**<0.001 ^U^**
**Spouse age (male)**	36 (32–41)	37 (33–42)	36 (32–40)	**<0.001 ^U^**
**Oocyte count**	9 (6–14)	9 (5–13)	10 (7–14)	**<0.001 ^U^**
**M2 oocyte count**	7 (5–11)	6 (4–10)	8 (5–11)	**<0.001 ^U^**
**Mature oocyte count**	0.82 (0.68–0.92)	0.82 (0.67–0.94)	0.82 (0.70–0.92)	0.468 ^U^
**Fertilization rate**	0.88 (0.78–1)	0.88 (0.75–1)	0.88 (0.78–1)	0.318 ^U^
**Transferred total embryo count**	2 (1–2)	2 (1–2)	1 (1–2)	**<0.001 ^U^**

^c^ Pearson Chi-Square Test (Monte Carlo), ^U^ Mann–Whitney U test (Monte Carlo), ^OR^ Odds Ratio (95% Confidence interval), Q1: 1st Quartile Q3: 3rd Quartile.

**Table 2:** Comparison of pregnancy successes of day 4 and day 5 transfers according to age, number of oocytes and number of embryos transferred.

		Transferred Total Embryo Count	Pregnancy	4th Day	5th Day	*p*
				n (%)	n (%)	
**Age ≤ 40**	**Oocyte count < 4**	**1**	Absent	53 (61.6)	22 (47.8)	0.143 ^c^
			Present	33 (38.4)	24 (52.2)	
		**2**	Absent	66 (47.8)	26 (40.0)	0.365 ^c^
			Present	72 (52.2)	39 (60.0)	
	**Total**		Absent	119 (53.1)	48 (43.2)	0.104 ^c^
			Present	105 (46.9)	63 (56.8)	
	**Oocyte count > 4**	**1**	Absent	144 (41.5)	375 (30.5)	**<0.001 ^c^**
			Present	203 (58.5)	853 (69.5)	1.6 (1.3–2.1) ^OR^
		**2**	Absent	185 (44.2)	284 (33.1)	**<0.001 ^c^**
			Present	234 (55.8)	575 (66.9)	1.6 (1.3–2.0) ^OR^
	**Total**		Absent	329 (43.0)	659 (31.6)	**<0.001 ^c^**
			Present	437 (57.0)	1428 (68.4)	1.6 (1.4–1.9) ^OR^
	**Total oocytes**	**1**	Absent	197 (45.5)	397 (31.2)	**<0.001 ^c^**
			Present	236 (54.5)	877 (68.8)	1.8 (1.5–2.3) ^OR^
		**2**	Absent	251 (45.1)	310 (33.5)	**<0.001 ^c^**
			Present	306 (54.9)	614 (66.5)	1.6 (1.3–2.0) ^OR^
	**Total**		Absent	448 (45.3)	707 (32.2)	**<0.001 ^c^**
			Present	542 (54.7)	1491 (67.8)	1.7 (1.5–2) ^OR^
**Age > 40**	**Oocyte count < 4**	**1**	Absent	35 (92.1)	11 (100.0)	0.999 ^f^
			Present	3 (7.9)	0 (0.0)	
		**2**	Absent	50 (64.9)	22 (48.9)	0.090 ^c^
			Present	27 (35.1)	23 (51.1)	
	**Total**		Absent	85 (73.9)	33 (58.9)	0.054 ^c^
			Present	30 (26.1)	23 (41.1)	
	**Oocyte count > 4**	**1**	Absent	7 (87.5)	8 (72.7)	0.603 ^c^
			Present	1 (12.5)	3 (27.3)	
		**2**	Absent	77 (74.0)	106 (53.3)	**0.001 ^c^**
			Present	27 (26.0)	93 (46.7)	2.5 (1.5–4.2) ^OR^
	**Total**		Absent	84 (75.0)	114 (54.3)	**<0.001 ^c^**
			Present	28 (25.0)	96 (45.7)	2.5 (1.5–4.2) ^OR^
	**Total oocytes**	**1**	Absent	42 (91.3)	19 (86.4)	0.673 ^c^
			Present	4 (8.7)	3 (13.6)	
		**2**	Absent	127 (70.2)	128 (52.5)	**<0.001 ^c^**
			Present	54 (29.8)	116 (47.5)	2.1 (1.4–3.2) ^OR^
	**Total**		Absent	169 (74.4)	147 (55.3)	**<0.001 ^c^**
			Present	58 (25.6)	119 (44.7)	2.4 (1.6–3.5) ^OR^

^c^ Pearson Chi-Square Test (Monte Carlo), ^f^ Fisher Exact Test(Monte Carlo), ^OR^ Odds Ratio (95% Confidence interval).

**Table 3:** Comparison of pregnancy successes of 4th- and 5th-day transfers according to the number of oocytes and the number of embryos transferred.

	Transferred Total Embryo Count	Pregnancy	4th Day	5th Day	*p*
			n (%)	n (%)	
**Oocyte count < 4**	1	Absent	88 (71.0)	33 (57.9)	0.091 ^c^
		Present	36 (29.0)	24 (42.1)	
	2	Absent	116 (54.0)	48 (43.6)	0.080 ^c^
		Present	99 (46.0)	62 (56.4)	
**Total**		Absent	204 (60.2)	81 (48.5)	**0.013 ^c^**
		Present	135 (39.8)	86 (51.5)	1.6 (1.1–2.3) ^OR^
**Oocyte count > 4**	1	Absent	151 (42.5)	383 (30.9)	**<0.001 ^c^**
		Present	204 (57.5)	856 (69.1)	1.7 (1.3–2.1) ^OR^
	2	Absent	262 (50.1)	390 (36.9)	**<0.001 ^c^**
		Present	261 (49.9)	668 (63.1)	1.7 (1.4–2.1) ^OR^
**Total**		Absent	413 (47.0)	773 (33.7)	**<0.001 ^c^**
		Present	465 (53.0)	1524 (66.3)	1.8 (1.5–2.1) ^OR^
**Total**	1	Absent	239 (49.9)	416 (32.1)	**<0.001 ^c^**
		Present	240 (50.1)	880 (67.9)	2.1 (1.7–2.6) ^OR^
	2	Absent	378 (51.2)	438 (37.5)	**<0.001 ^c^**
		Present	360 (48.8)	730 (62.5)	1.8 (1.5–2.1) ^OR^

^c^ Pearson Chi-Square Test (Monte Carlo), ^OR^ Odds Ratio (95% Confidence interval).

**Table 4:** Multiple logistic regression analysis results of embryo transfer on day 5 according to adjusted for age, oocyte count and transferred embryo count.

Adjusted for Age, Oocyte Count and Transferred Embryo Count						
Dependent Variable: Presence of Pregnancy	B	S.E.	*p*	Odds Ratio	95% C.I. for Odds Ratio	
					Lower	Upper
Day (5th day)	−0.662	0.071	<0.001	1.939	1.686	2.229
Constant	0.634	0.042	<0.001	1.885		

Multiple Logistic Regression (Method = Enter), C.I.: Confidence interval; B: regression coefficients; SE: Standard error.

## Data Availability

The data of the manuscript is deposited in the following URL address and is available on request. https://www.scidb.cn/en/s/vmEFBv (accessed on 7 October 2023).
